# Lack of Association of Variants Previously Associated with Anti-TNF Medication Response in Rheumatoid Arthritis Patients: Results from a Homogeneous Greek Population

**DOI:** 10.1371/journal.pone.0074375

**Published:** 2013-09-10

**Authors:** Maria I. Zervou, Efsevia Myrthianou, Irene Flouri, Darren Plant, Gregory Chlouverakis, Francesc Castro-Giner, Panayiota Rapsomaniki, Anne Barton, Dimitrios T. Boumpas, Prodromos Sidiropoulos, George N. Goulielmos

**Affiliations:** 1 Laboratory of Molecular Medicine and Human Genetics, Medical School of Crete, Heraklion, Crete, Greece; 2 Department of Rheumatology, Clinical Immunology and Allergy, Medical School of Crete, Heraklion, Greece; 3 NIHR Manchester Musculoskeletal Biomedical Research Unit, Manchester Academy of Health Sciences, Manchester, United Kingdom; 4 Department of Social Medicine, University of Crete, Heraklion, Greece; 5 Molecular and Population Genetics Laboratory, Wellcome Trust Centre for Human Genetics, University of Oxford, Oxford, United Kingdom; 6 Arthritis Research UK Epidemiology Unit, University of Manchester, Manchester, United Kingdom; 7 Medical School, National and Kapodistrian University of Athens, Athens, Greece; 8 Biomedical Research Foundation of the Academy of Athens, Athens, Greece; 9 Institute of Molecular Biology and Biotechnology, Foundation of Research and Technology–Hellas, Heraklion, Greece; Keio University School of Medicine, Japan

## Abstract

Treatment strategies blocking tumor necrosis factor (anti-TNF) have proven very successful in patients with rheumatoid arthritis (RA), showing beneficial effects in approximately 50-60% of the patients. However, a significant subset of patients does not respond to anti-TNF agents, for reasons that are still unknown. The aim of this study was to validate five single nucleotide polymorphisms (SNPs) of *PTPRC*, *CD226*, *AFF3*, *MyD88* and *CHUK* gene loci that have previously been reported to predict anti-TNF outcome. In addition, two markers of RA susceptibility, namely *TRAF1/C5* and *STAT4* were assessed, in a cohort of anti-TNF–treated RA patients, from the homogeneous Greek island of Crete, Greece. The RA patient cohort consisted of 183 patients treated with either of 3 anti-TNF biologic agents (infliximab, adalimumab and etanercept) from the Clinic of Rheumatology of the University Hospital of Crete. The SNPs were genotyped by TaqMan assays or following the Restriction Fragments Length Polymorphisms (RFLPs) approach. Disease activity score in 28 joints (DAS28) at baseline and after 6 months were available for all patients and analysis of good versus poor response at 6 months was performed for each SNP. None of the 7 genetic markers correlated with treatment response. We conclude that the gene polymorphisms under investigation are not strongly predictive of anti-TNF response in RA patients from Greece.

## Introduction

Rheumatoid Arthritis (RA) is an autoimmune disorder characterized by chronic and destructive inflammation in synovial joints, exhibiting a highly variable disease course [1]. Conventional disease-modifying antirheumatic drugs (DMARDs), most commonly methotrexate (MTX), remain the cornerstone of RA treatment [2]. However, patients for whom MTX produces an inadequate response are treated with biological agents which inhibits inflammatory cytokines (tumor necrosis factor (TNF)-α and interleukin 6 (IL-6) [3], deplete B-cells or inhibit T-cell activation. A major impediment to successful management is that 30–40% of patients have inadequate response and the reasons for this remain largely unknown. Anti-TNF agents work through the inhibition of the interaction between TNFα and its receptors, thus inhibiting the downstream signaling [4]. Five of these drugs, namely adalimumab, infliximab, etanercept, golimumab and certolizumab are approved for use in RA [2]. Ideally, clinicians would like to have predictors of response, in order to select the appropriate agent in individual basis. Reliable predictors (biomarkers, clinical predictors) are clinically important to avoid potential side effects from agents that will not have clinical benefit and will be an important first step as we move towards an era of stratified medicine [5].

Genetic markers could be useful in daily practice because they do not vary with time, and analysis can be carried out using samples derived from patient’s blood. Many pharmacogenetic studies dealing with anti-TNF response have included genes participating in various signaling pathways that regulate key immune and inflammatory processes. These studies were conducted following either the candidate-gene approach [6–11] or the emerging approach of genome-wide association studies (GWAS) [12]. Furthermore, research has focused on analyzing genes that are associated with the development of RA as potential predictors of anti-TNF efficacy [13–15]. Independent replication of findings in other populations will now be required to provide support that these associations are true positives. This study aimed to confirm whether five SNP markers, found in previous studies to predict responses to anti-TNF treatment of RA patients, are also associated with responses to therapy in a genetic homogeneous Greek population. Additionally, two genes that have previously been shown to correlate with RA development, namely *STAT4* rs7574865 and *TRAF1/C5* rs1081848 [16–18] were selected for investigation as putative markers of anti-TNF response due to the role of these genes in TNF signaling [19,20].

## Materials and Methods

Patients with RA who had received anti-TNF infusions in the Clinic of Rheumatology of the University Hospital of Crete, satisfying the American College of Rheumatology (ACR) criteria [21], were included (40 males, 143 females [78.1%]). TNFα blockade therapy was given to these patients following unsuccessful treatment with at least one disease modifying anti-rheumatic drug (DMARD). Clinical response was determined according to the European League Against Rheumatism (EULAR) criteria [22]. Information on age, gender, anti–cyclic citrullinated peptide (anti-CCP) antibody positivity, rheumatoid factor (RF), anti-TNF start and follow-up dates (treatment duration), DAS28 scores at baseline and at 6 months follow-up was recorded. The study was approved by the local Ethics Committee for medical research of the University Hospital of Heraklion, Crete, and was carried out in compliance with the declaration of Helsinki. Informed consent of the patients was oral because the samples were analyzed as anonymous ones and the aforementioned ethics committee approved this procedure.

We selected a panel of single nucleotide polymorphism (SNP) markers mapping to five recently suggested treatment-response associated genes (*PTPRC*, *CD226*, *AFF3*, *MyD88* and *CHUK*) and 2 RA susceptibility loci (*TRAF1-C5* and *STAT4*) for genotyping in a genetic homogeneous cohort of patients treated with anti-TNF agents. Allelic discrimination of the *PTPRC* rs10919563, *CD226* rs763361, *AFF3* rs10865035, *MyD88* rs7744 and *CHUK* rs11591741 SNPs was carried out using commercially available Taqman assays on a 7000 Real-Time PCR system (catalogue ID:C_31565763_10, C_1464836_20, C_2099360_10, C_3094830_10 and C_1345902_10, respectively) from Applied Biosystems (Foster City, CA, USA). Amplification and genotyping for the *TRAF1/C5* rs10818488 (*Sdu*I) and *STAT4* rs7574865 (*Hpa*I) was performed by restriction fragments lengths polymorphism by using protocols developed from our group and published previously [23]. Genotypes were scored blindly, and analysis of all ambiguous samples was repeated. Moreover, 10% of the samples were amplified twice for checking the accuracy of the results.

Summary descriptive statistics are presented as mean +/- SD, or frequencies, as appropriate. SNP variants and other categorical variables were compared between the responders and non-responders by means of chi-square or Fisher’s exact test. Continuous parameters were compared between these two groups with independent samples t-test. IBM-SPSS was used for the analyses, and all statistical tests were carried out at the two-sided 5% level of significance. Power analysis was conducted using the Quanto software (available at: http://hydra.usc.edu/gxe) at a two-sided level of p<0.05, assuming a log-additive genetic model, the sample size available, the observed allele frequencies in the general population and an estimated effect (odds ratio) of 1.2. No adjustment was made for anti-TNF drug type in these analyses, as the aim was to identify drug class effects.

## Results

The mean age at baseline this group of patients was 59.37±11.38 years, with 78.14% of patients being female. Radiographic erosions were found in 26.23% of cases. Concomitant DMARD (methotrexate, leflunomide, azathioprine or sulfasalazine) was given to 100% of the patients before changing the therapy to anti-TNF agents. The mean number of previous treatments, including corticosteroids (prednizolone), did not significantly differ between responders and non-responders. Patients were eligible for this study if they had not stopped or changed treatment during the last 6 months. Among the 183 patients included, 106 were responders and 77 non-responders. The clinical characteristics are summarized in Table 1. Differences in baseline characteristics were not significant between the two groups.

**Table 1:** Cohort characteristics of 183 RA patients treated with anti-TNF therapy.

	**Responders**	**Non-Responders**	**p-value**
Number	106	77	
Sex (% female)	74.5%	83.1%	0.205
Age, mean ± SD	59.26 ± 11.87	59.52 ± 10.72	0.881
RF positive	46.9%	45.9%	1.00
ACPA positive	59.43%	45.45%	0.231
Erosions	30%	20.6%	0.257
Start DAS, mean ± SD	6.22 ± 1.22	6.095 ± 1.47	0.524
End DAS, mean ± SD	4.05 ± 1.21	6.17 ± 1.29	<0.01
Improvement	2.18 ± 0.96	0.07 ± 0.97	<0.01
Infliximab treated %	73 (68.9%)	49 (63.6%)	0.526
Etanercept treated %	10 (9.4%)	13 (16.9%)	0.175
Adalimumab treated %	23 (21.7%)	15 (19.5%)	0.854
Number of previous biologic therapies, median (IQR)	1 (1-2)	1(1-1)	0.490
Methotrexate (MTX) usage	63.2%	55.8%	0.360
Mean dose of MTX (mg/week)	15.3 ± 3.6	13.8 ± 4.7	0.080
Prednisolone usage	20.8%	14.3%	0.330

Given the small number of our RA patients receiving anti-TNF agents, we combined “moderate” and “good” into “responders”. Thus, in our primary analysis, we included all patients. The frequency of the different genotypes, according to responder status is shown in Table 2. None of the SNPs under investigation was associated with response to the anti-TNF agents. The genotyping success for all the SNPs analyzed was >98%.

**Table 2:** Genetic association of seven SNPs with the response to treatment with anti-tumor necrosis factor agents in patients with RA from Crete.

Marker	Gene	Number		MAF		Genotype counts		p	OR (95% CI)
		Responders	Non-responders	Responders	Non-responders	Responders	Non-responders		
rs10919563	*PTPRC*	106	77	0.146	0.12	76/29/1	60/15/2	0.64	1.22 (0.54-2.72)
rs7574865	*STAT4*	106	77	0.24	0.30	55/51/0	32/44/1	0.23	1.345 (0.73-2.48)
rs10865035	*AFF3*	106	77	0.47	0.42	31/50/25	24/42/11	0.29	1.26 (0.72-2.18)
rs7744	*MyD88*	106	77	0.66	0.45	92/14/0	70/7/0	0.5	0.67 (0.19-2.29)
rs10818488	*TRAF1/ C5*	106	77	0.42	0.45	27/68/11	18/49/10	0.67	0.91 (0.52-1.57)
rs11591741	*CHUK*	106	77	0.37	0.32	42/49/15	36/33/8	0.32	1.273 (0.71-2.26)
rs763361	*CD226*	106	77	0.44	0.43	34/51/21	29/19/19	1	1.015 (0.58-1.76)

Since the clinical response of the RA patients to the first biologic agent is usually better than that to the second one, another analysis was conducted by using the 142 patients of this category (79 responders and 63 non-responders). Apart from a tendency of the rs7744 *MyD88* SNP to be associated with response to treatment, no genetic association was found for any of the SNPs examined.

In a second analysis, we categorized patients according to EULAR response criteria [22], i.e. where ‘good response’ was defined as ending DAS28<3.2 and DAS28 improvement from baseline > 1.2; ‘non-response’ was defined as DAS28 improvement<0.6 or improvement≤1.2, and ending DAS28>5.1. However, no association with treatment response was observed for any of the SNPs analyzed (data not shown). When a linear regression analysis was conducted by involving another clinical parameter, that is the DAS28 decrease rate (delta DAS28/original DAS28), no association with any of the 7 SNPs was detected either (data not shown).

## Discussion

The use of anti-TNF biological agents has transformed the management of RA, although a substantial proportion of treated patients demonstrate either partial or no response to these therapies. The present investigation is the first study of genetic predictors of anti-TNF response performed in Greece to date. The cohort of the Cretan RA patients analyzed in the present work is a part of the Hellenic Biologic Registry for Rheumatic Diseases that collects data from all patients who receive biologic agents in 7 Rheumatology Centers of Greece. Considering that independent replication is required to confirm definitively the association of SNPs with response to anti-TNF therapy, we performed the present study focusing on the genetic homogeneous population of Crete, which shares a common genetic and cultural background and a common environment, while it is characterized by good genealogical and clinical records and low migration rates, thus contributing to an increased reliability of the data collected [23–25]. However, our study failed to detect any association between seven SNPs and response to anti-TNF therapy.

SNPs that have been associated with response to anti-TNF therapy confer modest effects, thus, in order to detect subtle effects, studies require adequate power. The current study had 24% power to detect a difference in DAS28 of ≥ 0.6 (a clinically meaningful change) for allele frequencies of >5% (detailed power values are not presented). One possible explanation for our results may therefore be that our study is underpowered to detect modest effects. Of note, the allelic frequencies of the genes analyzed for association with drug response in the Cretan population are not markedly different than those in other European populations i.e. *PTPRC* MAF 0.15 in Crete vs. 0.12, *AFF3* 0.53 vs. 0.50, *CD226* 0.44 vs. 0.48, *STAT4* 0.24 vs. 0.25 [7,9,10]. These findings emphasize on the lack of any substantial genetic differences between Cretan and other European populations although various previous reports reported some distinct population-specific differences [26–28]. The two RA susceptibility loci analyzed, *TRAF1/C5* and *STAT4*, may also need further investigation. However, genes contributing to disease susceptibility are not necessarily associated with treatment response, as shown previously in the case of the well established RA susceptibility locus mapping to *PTPN22* [8]. Interestingly, another RA risk SNP of *TRAF1/C5*, the rs3761847, was found to be associated with anti-TNF treatment response in a Southern European population [29]. The rs 6427528 *CD84* SNP, which was reported recently that may serve as a useful biomarker for response to etanercept treatment in RA patients of European ancestry [30], will be also investigated in the Cretan population in an attempt to clarify its putative role in this cohort.

Despite substantial effort seen the last few years in the study of genetic markers of anti-TNF treatment response, only few associations have been identified [10]. Furthermore, the genetic associations reported previously are modestly effecting, in contrast, for example, to the large genetic effects seen in studies focusing on the role of *VKORC1* and *CYP2C9* genes with response to warfarin therapy [31]. It is therefore unlikely that markers of anti-TNF response with large effect exist and we will need to analyse thousands of individuals before reproducible finding will begin to be reported. This will require large national and international collaborations and sharing of datasets.

In conclusion, we present data that does not support any positive association between carriage of alleles for any of the seven SNPs examined and response to anti-TNF therapy in RA patients. However, larger studies are needed to definitively exclude the association of the SNPs under investigation in the Greek population.
